# *In Vitro* Antibiofilm Efficacies of Different Antibiotic Combinations with Zinc Sulfate against *Pseudomonas aeruginosa* Recovered from Hospitalized Patients with Urinary Tract Infection

**DOI:** 10.3390/antibiotics3010064

**Published:** 2014-02-17

**Authors:** Walid Elkhatib, Ayman Noreddin

**Affiliations:** 1 Department of Microbiology and Immunology, Faculty of Pharmacy, Ain Shams University, African Union Organization St. Abbassia, Cairo 11566, Egypt; E-Mail: walid.elkhatib@hamptonu.edu; 2 Department of Pharmacy Practice, School of Pharmacy, Hampton University, Kittrell Hall Hampton, Virginia 23668, USA; 3 Graduate Program of Biomedical Sciences, Eastern Virginia Medical School, 825 Fairfax Ave, Norfolk, Virginia 23507, USA

**Keywords:** biofilm, *Pseudomonas aeruginosa*, zinc sulfate, urinary tract infection, fluoroquinolone, synergism

## Abstract

Urinary tract infections (UTIs) are a serious healthcare dilemma influencing millions of patients every year and represent the second most frequent type of body infection. *P*seudomonas *aeruginosa* is a multidrug-resistant pathogen causing numerous chronic biofilm-associated infections including urinary tract, nosocomial, and medical devices-related infections. In the present study, the biofilm of *P*. *aeruginosa* CCIN34519, recovered from inpatients with UTIs, was established on polystyrene substratum and scanning electron microscopy (SEM) and was utilized for visualization of the biofilm. A previously described *in vitro* system for real-time monitoring of biofilm growth/inhibition was utilized to assess the antimicrobial effects of ciprofloxacin, levofloxacin, moxifloxacin, norfloxacin, ertapenem, ceftriaxone, gentamicin, and tobramycin as single antibiotics as well as in combinations with zinc sulfate (2.5 mM) against *P.* *aeruginosa* CCIN34519 biofilm. Meanwhile, minimum inhibitory concentrations (MICs) at 24 h and mutant prevention concentrations (MPCs) at 96 h were determined for the aforementioned antibiotics. The real-time monitoring data revealed diverse responses of *P.* *aeruginosa* CCIN34519 biofilm to the tested antibiotic-zinc sulfate combinations with potential synergisms in cases of fluoroquinolones (ciprofloxacin, levofloxacin, moxifloxacin, and norfloxacin) and carbapenem (ertapenem) as demonstrated by reduced MIC and MPC values. Conversely, considerable antagonisms were observed with cephalosporin (ceftriaxone) and aminoglycosides (gentamicin, and tobramycin) as shown by substantially increased MICs and MPCs values. Further deliberate *in vivo* investigations for the promising synergisms are required to evaluate their therapeutic potentials for treatment of UTIs caused by *P. aeruginosa* biofilms as well as for developing preventive strategies.

## 1. Introduction

Biofilm associated infections tend to be reluctant and difficult to eradicate [1]. The increased antibiotic resistance of biofilms was generally attributed to the biofilm-associated patterns of gene expression, slow growth rate, and substantially diminished antimicrobial diffusion within the biofilm [2]. Biofilms are considered relevant to the clinical settings because they play key roles in the ability of biofilm-ensconced bacteria to tolerate relatively high therapeutic doses of antibiotics and persist in chronic infections [3,4]. Urinary tract infections (UTIs) are one of the most common bacterial infections affecting humans all through their life period [5,6]. UTIs are responsible for more than eight million visits to clinicians and more than two million admissions to the emergency rooms in the United States annually [6,7]. Furthermore, UTIs are the most common urological disease in the United States, with a financial burden on the healthcare system exceeding $3.5 billion annually [8].

*Pseudomonas aeruginosa* is an important opportunistic human pathogen and is eminent for chronic infections including medical device-associated infections as well as urinary tract infections [9]. The urinary tract infection (UTI) caused by *P. aeruginosa* is a serious health problem affecting millions of people worldwide each year and catheterization of the urinary tract is one of the most common predisposing factors to such infections [6]. Indisputably, biofilm formation was considered to be the key feature of *P.* *aeruginosa* survival in chronic infections, and extracellular matrices of the biofilms provide structural scaffold and protective barricade against antibiotics [10,11]. In *P. aeruginosa* biofilm, the efficacy of the antimicrobial chemotherapy may be attenuated not only by deficiency of antibiotics access caused by the sheltered bacteria within the exopolysaccharide alginate matrix but also by emergence of the mutant subpopulations [12].

Fluoroquinolones, β-lactams and aminoglycosides are the main classes of antibiotics used for treatment of *P. aeruginosa* infections. Unfortunately, the multi-drug resistant isolates of *P. aeruginosa* which patients are often exposed to in hospital settings are resistant to one or more of these antibiotic classes [13,14,15,16,17]. Selective pressure due to excessive exposure of bacteria to antibiotics is generally the origin of such high incidence of resistance in the hospitals environment. Intensive care units (ICU) and long-term care facilities are also notorious worldwide for harboring multi-drug and pan-drug resistant *P. aeruginosa* strains [17,18]. Owing to the unmet medical needs of novel antimicrobial agents [19,20], the use of combination therapy has gained attention as an option strategy for combating *P. aeruginosa* biofilm associated infections [21,22,23]. Zinc is a vital trace element required for virtually all forms of life [24]. It is also essential for all bacteria, but surplus amounts of the metal can possess toxic effects on them [25] and zinc chelation with nitroxoline, a urinary antibiotic, induced the dispersal of *P. aeruginosa* biofilms [26]. In this context, the objective of the current study was to evaluate the antimicrobial interactions of different fluoroquinolones, carbapenem, cephalosporin, and aminoglycosides with zinc sulfate against biofilm of the uro-pathogen, *P.* *aeruginosa* CCIN34519, through implementation of *in vitro* real-time monitoring system.

## 2. Results and Discussion

### 2.1. Scanning Electron Microscopy of the Biofilm

**Figure 1:**
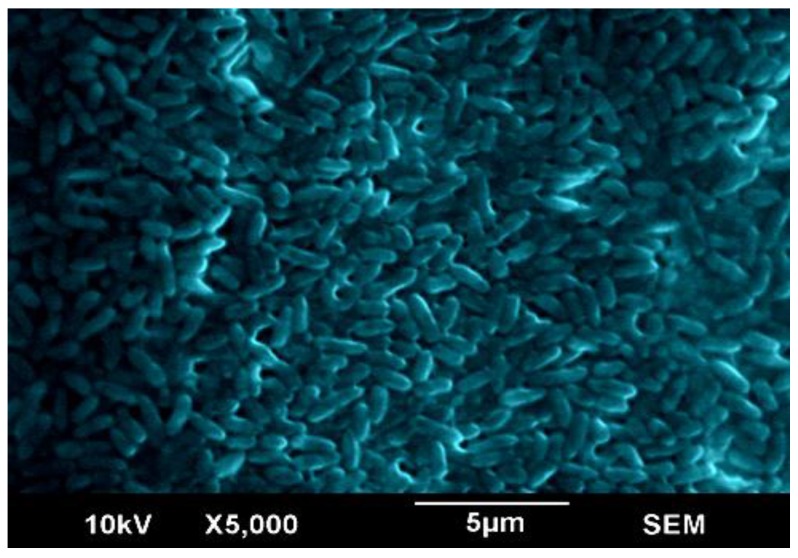
Colored scanning electron micrograph illustrating the appearance of *P. aeruginosa* CCIN34519 biofilm on polystyrene substratum.

Variable pressure scanning electron microscopy was used for visualization of the biofilm on polystyrene plates after 12 h of incubation in absence of physical distortion or gold coating of the biofilm. The SEM demonstrated a well-established *P.* *aeruginosa* CCIN34519 biofilm as shown in Figure 1.

### 2.2. Real-Time Monitoring of P. aeruginosa CCIN34519 Biofilm

**Figure 2:**
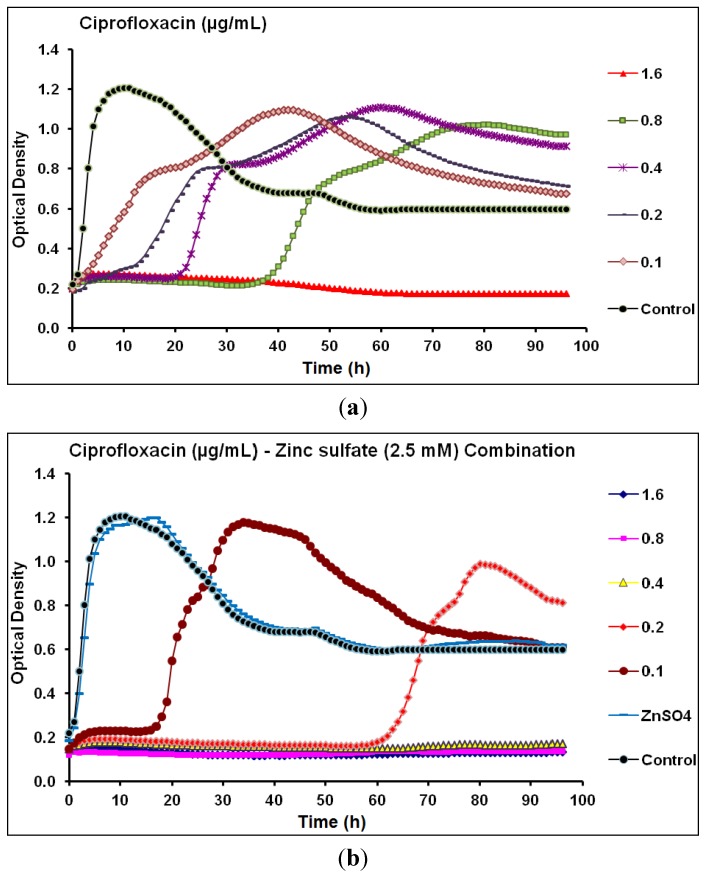
Real-time data showing the effect of (**a**) Ciprofloxacin alone (0.1–1.6 µg/mL) and (**b**) Ciprofloxacin (0.1–1.6 µg/mL) in combination with zinc sulfate (2.5 mM) on *P*. *aeruginosa* CCIN34519 biofilm as monitored by the Bioscreen C over 96 h. Control represents cation adjusted Mueller Hinton II broth (CAMH) while, ZnSO_4_ indicates CAMH broth supplemented with 2.5 mM zinc sulfate.

The effect of different concentrations of ciprofloxacin (0.1–1.6 µg/mL) alone on *P.* *aeruginosa* CCIN34519 biofilm as compared to the control (cation adjusted Mueller Hinton II; CAMH broth) is shown in Figure 2a. The minimum inhibitory concentration (MIC) and mutant prevention concentration (MPC) of ciprofloxacin against *P.* *aeruginosa* CCIN34519 biofilm, as determined by kinetic measurements by means of the Bioscreen C, were 0.8 µg/mL at 24 h and 1.6 µg/mL at 96 h, respectively. Real-time monitoring data showed that ciprofloxacin alone inhibited the biofilm growth for 4, 8, 20, and 38 h with the tested concentration levels of 0.1, 0.2, 0.4, and 0.8 µg/mL, respectively. For the untreated biofilm, microbial growth from *P.* *aeruginosa* CCIN34519 biofilm exponentially increased and reached the peak at 10 h of incubation, followed by a gradual decline for up to 35 h, and finally maintained the plateau until the end of the experiment (Figure 2a). In this study, zinc sulfate concentration (2.5 mM) was selected as the highest concentration that had no inhibitory effect on the growth of *P.* *aeruginosa* CCIN34519 biofilm as shown in Figure 2b. In the presence of zinc sulfate (2.5 mM), the MIC and MPC of ciprofloxacin against *P.* *aeruginosa* CCIN34519 biofilm decreased to 0.2 µg/mL at 24 h and 0.4 µg/mL at 96 h, respectively. Furthermore, ciprofloxacin could inhibit the biofilm growth for 17 and 60 h at concentration levels of 0.1, and 0.2 µg/mL, respectively (Figure 2b).

**Figure 3:**
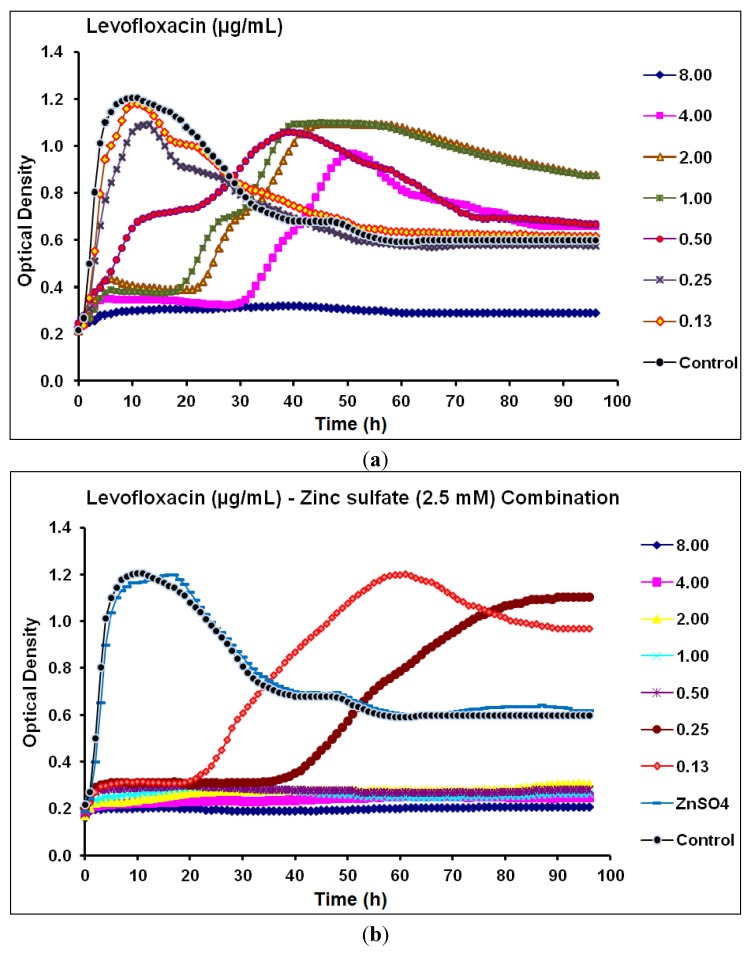
Real-time data showing the effect of (**a**) Levofloxacin alone (0.13–8.00 µg/mL) and (**b**) Levofloxacin (0.13–8.00 µg/mL) in combination with zinc sulfate (2.5 mM) on *P*. *aeruginosa* CCIN34519 biofilm as monitored by the Bioscreen C over 96 h. Control represents cation adjusted Mueller Hinton II broth (CAMH) while, ZnSO_4_ indicates CAMH broth supplemented with 2.5 mM zinc sulfate.

The MIC and MPC of levofloxacin against *P.* *aeruginosa* CCIN34519 biofilm were 4.0 µg/mL and 8.0 µg/mL, respectively, and levofloxacin alone inhibited the biofilm growth for 18, 22, and 30 h with the concentration levels of 1.0, 2.0, and 4.0 µg/mL, respectively (Figure 3a). On the other hand, the MIC and MPC of levofloxacin, in the presence of zinc sulfate (2.5 mM), were curtailed to 0.25 µg/mL and 0.5 µg/mL, respectively. Furthermore, levofloxacin inhibited the biofilm growth for 20 and 36 h at concentration levels of 0.13, and 0.25 µg/mL, respectively (Figure 3b).

**Figure 4:**
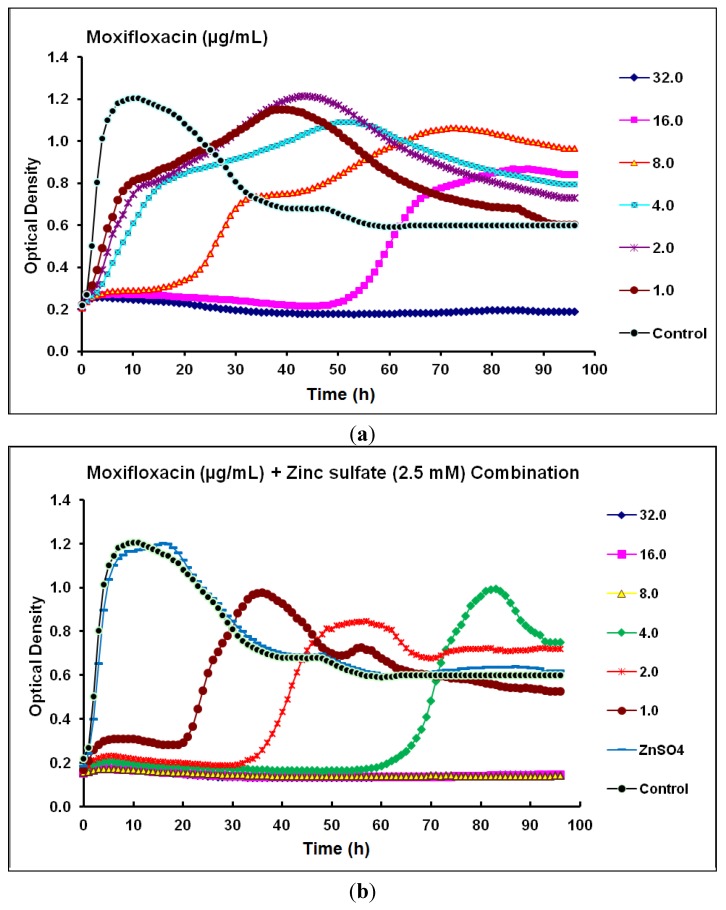
Real-time data showing the effect of (**a**) Moxifloxacin alone (1.0–32.0 µg/mL) and (**b**) Moxifloxacin (1.0–32.0 µg/mL) in combination with zinc sulfate (2.5 mM) on *P*. *aeruginosa* CCIN34519 biofilm as monitored by the Bioscreen C over 96 h. Control represents cation adjusted Mueller Hinton II broth (CAMH) while, ZnSO_4_ indicates CAMH broth supplemented with 2.5 mM zinc sulfate.

For moxifloxacin, the MIC and MPC of against *P.* *aeruginosa* CCIN34519 biofilm, as determined by kinetic measurements, were 16 µg/mL and 32 µg/mL, respectively. Real-time monitoring data demonstrated that moxifloxacin alone suppressed the biofilm growth for 15 and 50 h with the tested concentration levels of 8 and 16 µg/mL, respectively (Figure 4a). In the presence of zinc sulfate (2.5 mM), the MIC and MPC of moxifloxacin against *P.* *aeruginosa* CCIN34519 biofilm reduced to 2.0 µg/mL and 8.0 µg/mL, respectively. Furthermore, moxifloxacin could inhibit the biofilm growth for up to 20, 32 and 58 h at concentration levels of 1.0, 2.0 and 4.0 µg/mL, respectively (Figure 4b).

**Figure 5:**
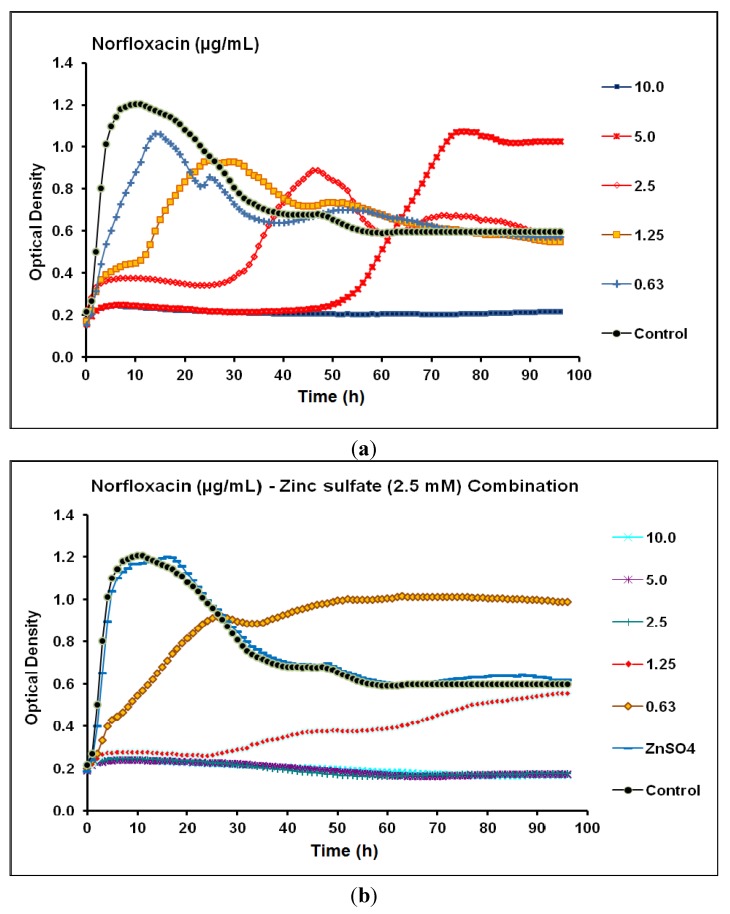
Real-time data showing the effect of (**a**) Norfloxacin alone (0.63–10.0 µg/mL) and (**b**) Norfloxacin (0.63–10.0 µg/mL) in combination with zinc sulfate (2.5 mM) on *P*. *aeruginosa* CCIN34519 biofilm as monitored by the Bioscreen C over 96 h. Control represents cation adjusted Mueller Hinton II broth (CAMH) while, ZnSO_4_ indicates CAMH broth supplemented with 2.5 mM zinc sulfate.

Regarding norfloxacin, the MIC and MPC against *P.* *aeruginosa* CCIN34519 biofilm were 5.0 µg/mL and 10.0 µg/mL, respectively. Norfloxacin suppressed the biofilm slightly above the baseline and completely at the baseline for 28 and 47 h at concentration levels of 2.5 and 5.0 µg/mL, respectively and its lower concentrations (0.63–1.25 µg/mL) could not suppress the biofilm (Figure 5a). On the other hand, the MIC and MPC of norfloxacin, in the presence of zinc sulfate (2.5 mM), diminished to 1.25 µg/mL and 2.5 µg/mL, respectively. Furthermore, norfloxacin inhibited the biofilm growth for 26 and 96 h at concentration levels of 1.25 and 2.5 µg/mL, respectively (Figure 5b).

**Figure 6:**
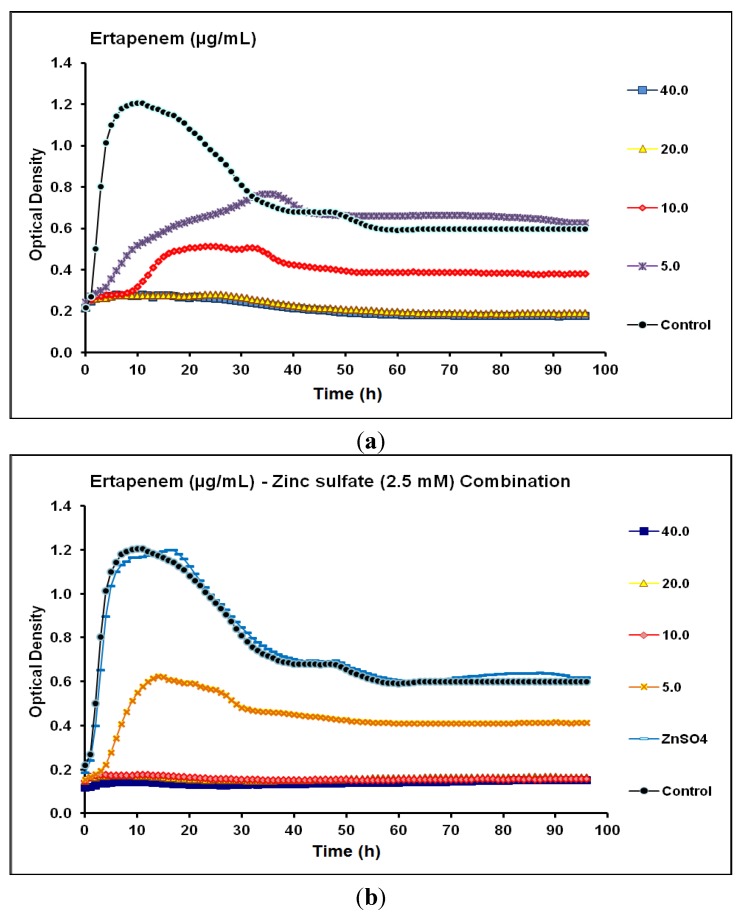
Real-time data showing the effect of (**a**) Ertapenem alone (5.0–40.0 µg/mL) and (**b**) Ertapenem (5.0–40.0 µg/mL) in combination with zinc sulfate (2.5 mM) on *P*. *aeruginosa* CCIN34519 biofilm as monitored by the Bioscreen C over 96 h. Control represents cation adjusted Mueller Hinton II broth (CAMH) while, ZnSO_4_ indicates CAMH broth supplemented with 2.5 mM zinc sulfate.

The MIC and MPC of ertapenem against *P.* *aeruginosa* CCIN34519 biofilm maintained the same value of 20 µg/mL. Real-time data demonstrated that ertapenem alone suppressed the biofilm growth for 5 and 10 h at concentration levels of 5 and 10 µg/mL, respectively. Moreover, both concentrations markedly abridged the biofilm growth for up to 30 h and the biofilm maintained a prominent low level of microbial growth from 30–96 h of incubation with ertapenem (10 µg/mL) as compared to the control (Figure 6a). In the presence of zinc sulfate (2.5 mM), both MIC and MPC of ertapenem against *P.* *aeruginosa* CCIN34519 biofilm reduced to 10 µg/mL. Furthermore, ertapenem inhibited the biofilm growth for up to 5 and 96 h at concentration levels of 5 and 10 µg/mL, respectively and the biofilm showed a marked reduction of the microbial growth over 96 h with ertapenem (5 µg/mL) as compared to the control (Figure 6b).

**Figure 7:**
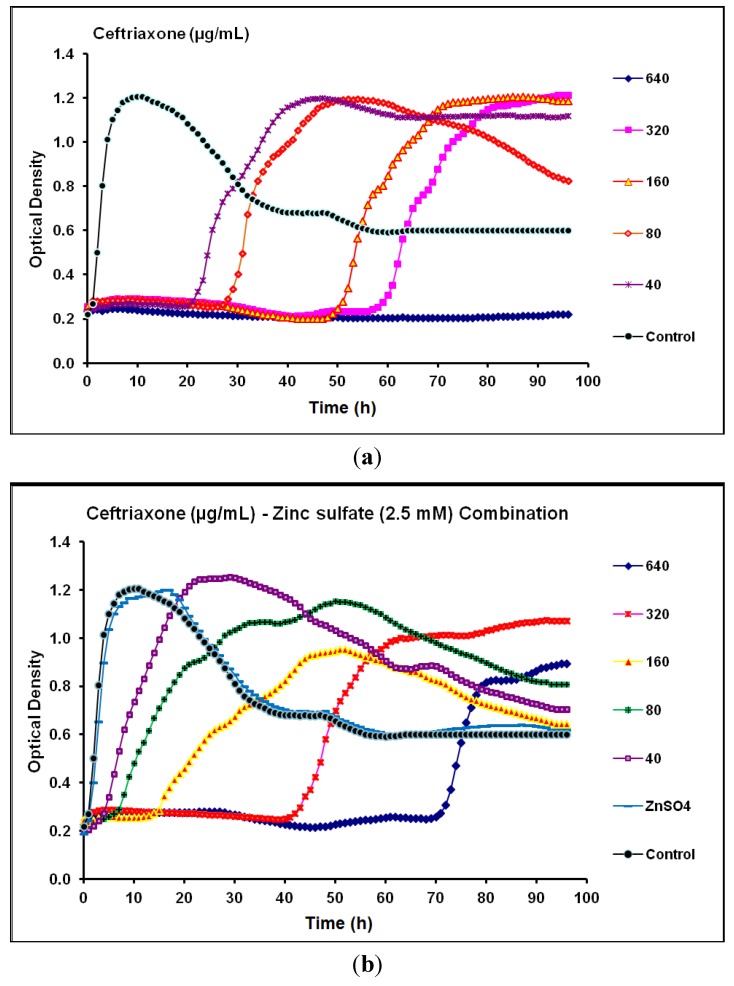
Real-time data showing the effect of (**a**) Ceftriaxone alone (40–640 µg/mL) and (**b**) Ceftriaxone (40–640 µg/mL) in combination with zinc sulfate (2.5 mM) on *P*. *aeruginosa* CCIN34519 biofilm as monitored by the Bioscreen C over 96 h. Control represents cation adjusted Mueller Hinton II broth (CAMH) while, ZnSO_4_ indicates CAMH broth supplemented with 2.5 mM zinc sulfate.

Concerning ceftriaxone, the MIC and MPC against *P.* *aeruginosa* CCIN34519 biofilm were 80 µg/mL and 640 µg/mL, respectively. Depending on the kinetic data, ceftriaxone alone inhibited the biofilm growth for 20, 28, 48, and 57 h with the tested concentration levels of 40, 80, 160, and 320 µg/mL, respectively (Figure 7a). Conversely, the MIC and MPC of ceftriaxone, in presence of zinc sulfate (2.5 mM), considerably increased to 320 µg/mL and >640 µg/mL, respectively. Additionally, ceftriaxone-zinc sulfate (2.5 mM) combination could inhibit the biofilm growth for up to 5, 8, 16, 40, and 72 h at concentration levels of 40, 80, 160, 320, and 640 µg/mL, respectively, but none of the tested ceftriaxone concentrations could suppress *P.* *aeruginosa* CCIN34519 biofilm over 96 h (Figure 7b).

**Figure 8:**
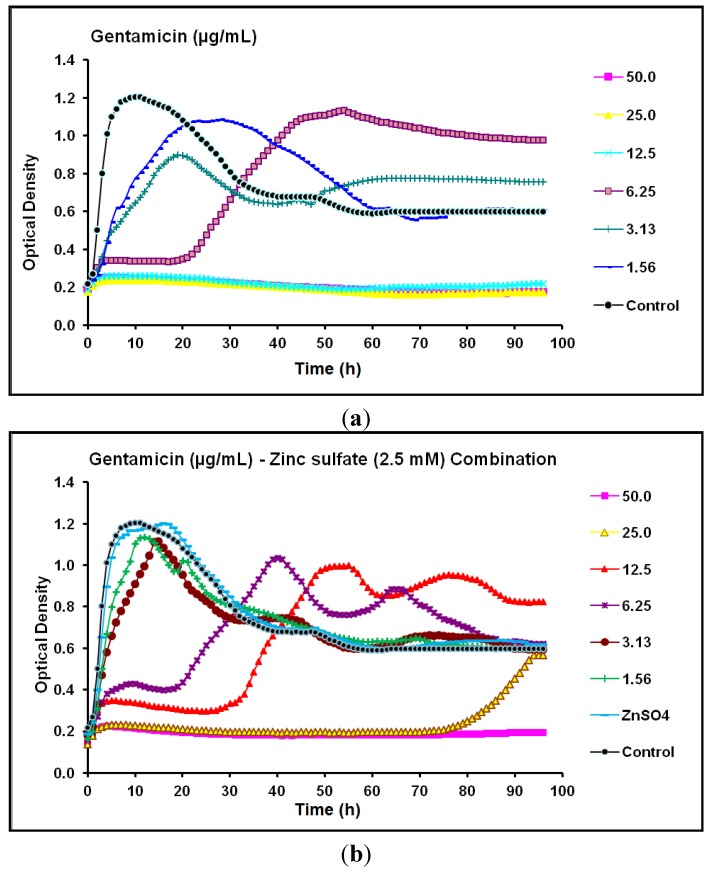
Real-time data showing the effect of (**a**) Gentamicin alone (1.56–50.0 µg/mL) and (**b**) Gentamicin (1.56–50.0 µg/mL) in combination with zinc sulfate (2.5 mM) on *P*. *aeruginosa* CCIN34519 biofilm as monitored by the Bioscreen C over 96 h. Control represents cation adjusted Mueller Hinton II broth (CAMH) while, ZnSO_4_ indicates CAMH broth supplemented with 2.5 mM zinc sulfate.

With gentamicin, the MIC and MPC against *P.* *aeruginosa* CCIN34519 biofilm maintained the same value of 12.5 µg/mL. Gentamicin suppressed the biofilm to some extent above the baseline and entirely at the baseline for 20 and 96 h at concentration levels of 6.25 and 12.5 µg/mL, respectively and its lower concentrations (1.56–3.13 µg/mL) could not suppress the biofilm (Figure 8a). On the contrary, the MIC and MPC of gentamicin, in presence of zinc sulfate (2.5 mM), substantially increased to 25 µg/mL and 50 µg/mL, respectively. Furthermore, gentamicin partially suppressed the biofilm growth, parallel to the baseline at 6.25 and 12.5 µg/mL for 18 and 30 h, respectively, but completely inhibited it at 25 µg/mL for 75 h. Lower concentrations of gentamicin (1.56–3.13 µg/mL) in combination with zinc sulfate (2.5 mM) could not inhibit the biofilm and demonstrated higher growth rate over the initial 10 h of the incubation period as compared to that of gentamicin alone (Figure 8b).

**Figure 9:**
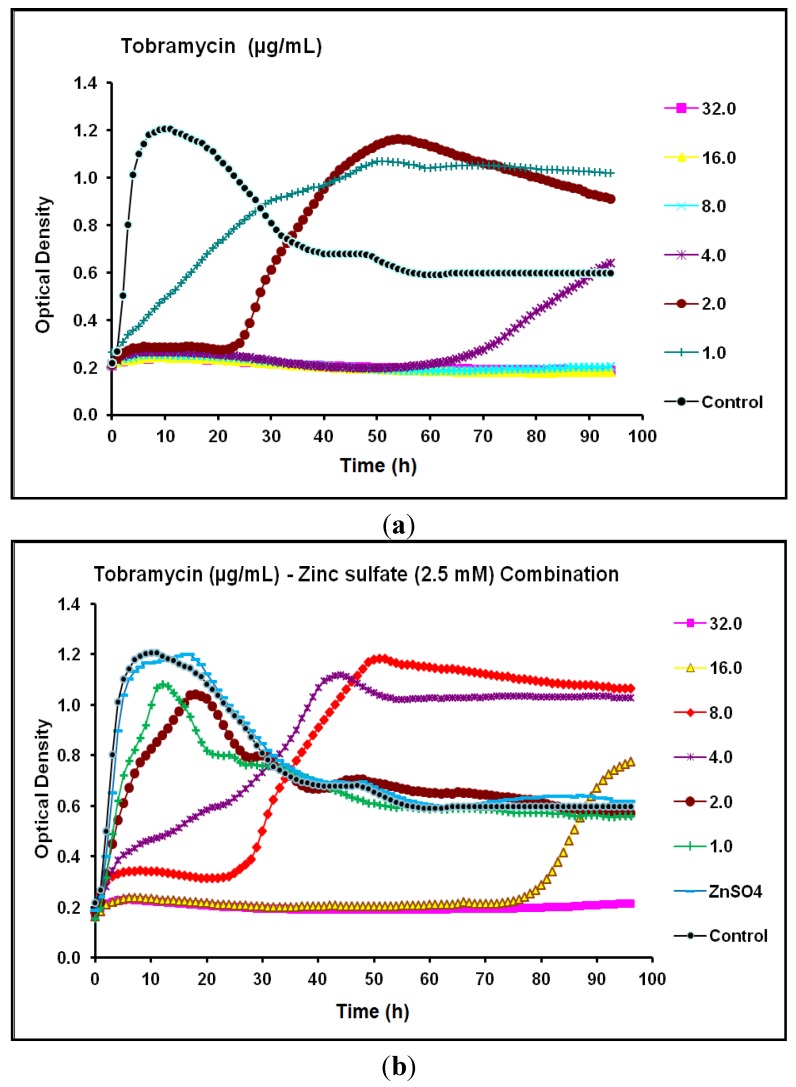
Real-time data showing the effect of (**a**) Tobramycin alone (1.0–32.0 µg/mL) and (**b**) Tobramycin (1.0–32.0 µg/mL) in combination with zinc sulfate (2.5 mM) on *P*. *aeruginosa* CCIN34519 biofilm as monitored by the Bioscreen C over 96 h. Control represents cation adjusted Mueller Hinton II broth (CAMH) while, ZnSO_4_ indicates CAMH broth supplemented with 2.5 mM zinc sulfate.

Regarding tobramycin, the MIC and MPC against *P.* *aeruginosa* CCIN34519 biofilm were 4.0 µg/mL and 8.0 µg/mL, respectively. Tobramycin alone could inhibit the biofilm growth for 22 and 64 h at concentration levels of 2.0 and 4.0 µg/mL, respectively (Figure 9a). Quite the opposite, the MIC and MPC of tobramycin, in the presence of zinc sulfate (2.5 mM), noticeably increased to 16 µg/mL and 32 µg/mL, respectively. Moreover, tobramycin partially suppressed the biofilm growth at 8.0 µg/mL for 22 h and completely inhibited it at 16 µg/mL for 76 h. Similar to gentamicin, lower concentrations of tobramycin (1.0–4.0 µg/mL) in combination with zinc sulfate (2.5 mM) could not inhibit the biofilm and showed a higher growth rate over the initial 12 h of the incubation period as compared to that of tobramycin alone (Figure 9b).

The synergistic and antagonistic effects of zinc sulfate (2.5 mM) on different tested antibiotics against *P. aeruginosa* CCIN34519 biofilm are summarized in Table 1.

**Table 1:** Effects of zinc sulfate on different antibiotics minimum inhibitory concentrations (MICs) and mutant prevention concentrations (MPCs) against *P. aeruginosa* CCIN34519 biofilm.

Antibiotic	Class	Biofilm Without Zinc Sulfate		Biofilm With Zinc Sulfate *		Anti-biofilm Efficacy of Combination
		MIC (24 h)	MPC (96 h)	MIC (24 h)	MPC (96 h)	
**Ciprofloxacin**	Fluoroquinolone	0.8	1.6	0.20	0.40	Synergistic
**Levofloxacin**	Fluoroquinolone	4.0	8.0	0.25	0.50	Synergistic
**Moxifloxacin**	Fluoroquinolone	16	32	2.0	8.0	Synergistic
**Norfloxacin**	Fluoroquinolone	5.0	10	1.25	2.5	Synergistic
**Ertapenem**	Carbapenem	20	20	10	10	Synergistic
**Ceftriaxone**	Cephalosporin	80	640	320	> 640	Antagonistic
**Gentamicin**	Aminoglycoside	12.5	12.5	25	50	Antagonistic
**Tobramycin**	Aminoglycoside	4.0	8.0	16	32	Antagonistic

* Zinc sulfate concentration (2.5 mM); MIC = Minimum Inhibitory Concentration; MPC = Mutant Prevention Concentration.

According to the MICs interpretive standards stated by Clinical and Laboratory Standards Institute (CLSI) [27], *P. aeruginosa* CCIN34519 biofilm were susceptible to ciprofloxacin and tobramycin; of intermediate resistance to levofloxacin, norfloxacin and gentamicin; and resistant to moxifloxacin, ertapenem and ceftriaxone. Concerning MPC at 96 h, *P. aeruginosa* CCIN34519 biofilm was considered to demonstrate intermediate resistance to ciprofloxacin, norfloxacin, gentamicin and tobramycin; and be resistant to levofloxacin, moxifloxacin, ertapenem and ceftriaxone. In the presence of zinc sulfate (2.5 mM), *P. aeruginosa* CCIN34519 biofilm was susceptible to ciprofloxacin, levofloxacin, moxifloxacin and norfloxacin; demonstrate intermediate resistance to ertapenem; and be resistant to ceftriaxone, gentamicin, and tobramycin. On the other hand, at 96 h and in combination with zinc sulfate (2.5 mM), *P. aeruginosa* CCIN34519 biofilm maintained its susceptibilities to ciprofloxacin, levofloxacin and norfloxacin; revealed intermediate resistance to moxifloxacin, and ertapenem; and showed resistance to ceftriaxone, gentamicin, and tobramycin.

### 2.3. Discussion

Bacterial biofilms represent global and predominant causes of both chronic infections and indwelling medical devices associated infections such as catheters and prostheses. Such infections typically exhibit significantly enhanced resistance to antimicrobial agents rendering them challenging to treat using conventional chemotherapeutic agents [28]. Standard doses of the antibiotic therapy can reduce the biofilm but rarely eradicates the entire biofilm [29,30]. Some theories attributed the antimicrobial resistance of the biofilm to its physical architecture, mutations, and altered gene expression patterns [31,32,33]. *P.* *aeruginosa* is one of the predominant causes of UTIs, nosocomial and intensive care unit-associated infections that occur annually worldwide [6,34]. As a consequence of biofilm formation, frequency of the resistant *P.* *aeruginosa* isolates present in clinical settings has been radically increased, thereby mandating the development of alternative therapeutic strategies [35]. In this context, *P. aeruginosa* biofilms have demonstrated resistance to different classes of antibiotics including ciprofloxacin (fluoroquinolone), ceftazidime (cephalosporin), and tobramycin (aminoglycoside) at concentrations far superior to the therapeutically achievable concentrations [36,37] and one hypothesis explaining biofilm resistance proposes the existence of the persister cells within the biofilm which are metabolically inactive and consequently are not destroyed by antimicrobial agents [38,39,40,41].

In the current study, the results of the SEM revealed that *P. aeruginosa* CCIN34519 biofilm could extensively colonize the polystyrene substratum within 12 h of incubation as well as to develop a relatively uniform architecture. Some investigators reported that a thick *P. aeruginosa* biofilm on surface of the polyethylene tubing was observed by SEM at 48 h in their *in vivo* experiments and repeatable pattern of cell death and lysis has been shown in *P. aeruginosa* biofilm during its development [42]. The difference in time of the biofilm establishment may be attributed to the experimental design and strain difference as well as nature of the used substrata. Main factors influencing the biofilm development may include temperature, nutrient composition, aeration, flow rate, and history of the cultures used for the inoculation, thus the experimental reproducibility can be extensively improved if these factors have been considered [43]. In the present study, the above mentioned variables were efficiently controlled in the conducted experiments through utilizing the Bioscreen C technology. 

It has been demonstrated that *P.* *aeruginosa* isolates were more resistant to growth inhibition by antibiotics when grown in biofilm cultures than when grown in planktonic ones [44,45]. For that reason, the failure of conventional planktonic culture techniques to predict antibiotic susceptibilities may explain inability of the antimicrobial agents to eradicate *P. aeruginosa* biofilm-associated UTIs. In view of that, all the experiments in this study were carried on the biofilm of *P.* *aeruginosa* CCIN34519 strain recovered from hospitalized patients with urinary tract infection through NAUTICA study [46].

It was previously reported that zinc oxide showed antibacterial and antifungal activity against planktonic cultures of some tested micro-organisms in a concentration dependent manner [47]. Moreover, complexed zinc with protoporphyrin IX or mesoprotoporphyrin IX were both highly effective in negating planktonic growth and biofilm formation of some tested bacteria and these zinc complexes act as iron siderophore analogs by supplanting the natural iron uptake of the tested bacteria [48]. *P. aeruginosa* regulates multiple genes by the zinc-dependent DksA transcriptional regulator at low zinc conditions. Nevertheless, excess amounts of this trace element can be potentially lethal for the bacteria. Thus, most bacteria have developed systems for both zinc acquisition and detoxification [25]. Concerning the MIC values in this study, zinc sulfate (2.5 mM) could increase the efficacies of ertapenem, ciprofloxacin, levofloxacin, moxifloxacin, and norfloxacin by 2, 4, 16, 8, and 4 folds, respectively, but decreased the efficacies of ceftriaxone, gentamicin, and tobramycin by 4, 2, and 4 folds, respectively, on *P. aeruginosa* CCIN34519 biofilm. Previous studies mentioned that doses of the antibiotics required to inhibit or eradicate the established biofilms frequently exceed the maximum achievable plasma concentrations for such antibiotics [44,45] and retention of the antibiotics in the anti-pseudomonal cache is a critical issue throughout the world as there is a limited number of effective drugs for treatment of *P. aeruginosa* infections [49]. Generally, fluoroquinolones have a broad spectrum of antimicrobial coverage and ciprofloxacin and, in particular, is one of the relatively effective antibiotics against *P.* *aeruginosa* infections in clinical settings [50,51]. Although *P. aeruginosa* CCIN34519 biofilm revealed no susceptibility at 96 h to all the tested antibiotics, the biofilm maintained its susceptibility to three fluoroquinolones (ciprofloxacin [MPC = 0.4 µg/mL], levofloxacin [MPC = 0.5 µg/mL], and norfloxacin [MPC = 2.5 µg/mL]) out of the eight tested antibiotics in the presence of zinc sulfate (2.5 mM) reserving such drugs as effective agents and within the therapeutically achievable concentrations for treatment of *P. aeruginosa* CCIN34519 biofilm in UTIs.

Prior to mutant selection or acquisition of exogenous resistance, *P. aeruginosa* possesses powerful efflux pumps to eliminate almost all classes of antibiotics [49,52] and the concept of its being a super bug was built on such a foundation [13]. The reported MPCs for some fluoroquinolones against *P. aeruginosa* ranged from 2 to >32.0 µg/mL depending on the study and the bacterial genotype. Nevertheless, the first-step *par C* gene mutation dramatically increases the MPC far above the original MPC [53,54,55,56]. Consistent with these findings, MPCs of the tested fluoroquinolones in this study ranged from 1.6–32.0 µg/mL against *P. aeruginosa* CCIN34519 biofilm. On the other hand, range of the MPCs for such fluoroquinolones prominently decreased to 0.4–8.0 µg/mL in combination with zinc sulfate (2.5 mM). In presence of zinc sulfate (2.5 mM), real-time data revealed that the tested fluoroquinolones (ciprofloxacin, levofloxacin, moxifloxacin, and norfloxacin) at lower concentrations (0.2, 0.25, 4.0, and 1.25 µg/mL, respectively) could suppress the growth of *P. aeruginosa* CCIN34519 biofilm for longer periods of time (60, 36, 58, and 26 h, respectively) and ertapenem (5.0 µg/mL) noticeably reduced the biofilm growth as compared to such antibiotics alone at the same concentration levels. Consequently, these antibiotics may afford better antimicrobial coverage against *P. aeruginosa* biofilm for extended duration and within the same therapeutic doses in case of concomitant administrations with zinc sulfate.

The potential for serious antagonistic effects by zinc sulfate (2.5 mM) combination with cephalosporin (ceftriaxone) or aminoglycosides (gentamicin and tobramicin) underscores the inevitability to circumvent the concomitant administration of over the counter (OTC) zinc sulfate containing preparations with the prescribed cephalosporins and aminoglycosides during the therapy of *P. aeruginosa* biofilm related infections such as nosocomial catheter-associated UTI as well as soft tissue infections. In the current study, the observed antagonism by zinc sulfate combination with gentamicin and tobramicin against *P. aeruginosa* CCIN34519 biofilm may be attributed to general interference of the divalent cations with the uptake of the aminoglycosides at both the outer and inner membranes in *P. aeruginosa* [57]. On the other hand, zinc sulfate (2.5 mM) demonstrated synergistic and antagonistic effects in combination with ertapenem and ceftriaxone, respectively. These paradoxical effects with the tested β-lactam antibiotics may be explained by the variable zinc affinities to different metallo-beta-lactamases [58]. Fortunately, the UTI patients infected with *P. aeruginosa* biofilm may possess an edge in overcoming the infection when ertapenem or fluoroquinolones (ciprofloxacin, levofloxacin, moxifloxacin, and norfloxacin) have been prescribed with simultaneous administration with zinc sulfate-containing preparations and such cases should be deliberately investigated.

## 3. Experimental

### 3.1. Bacterial Strain and Antimicrobial Agents

*P. aeruginosa* CCIN34519 strain used in this study was recovered from hospitalized patients with urinary tract infection through North American Urinary Tract Infection Collaborative Alliance (NAUTICA) study [46]. Before each experiment, *P. aeruginosa* CCIN34519 was sub-cultured on Mueller Hinton agar and incubated for 18–20 h at 37 °C. The inoculum was then prepared in cation-adjusted Mueller Hinton II broth (CAMH) and diluted to match 0.5 McFarland standard, which is equivalent to 1.5 × 10^8^ CFU/mL. Ceftriaxone sodium, ciprofloxacin hydrochloride, gentamicin sulfate, levofloxacin, moxifloxacin hydrochloride, norfloxacin, tobramycin, and zinc sulfate heptahydrate were purchased from Sigma-Aldrich (St. Louis, MO, USA). Ertapenem disodium powder was obtained from Thermo-Fisher Scientific (Waltham, MA, USA). All antibiotic stock solutions and dilutions were prepared according to the Clinical and Laboratory Standards Institute (CLSI) guidelines [27]. Stock solutions of the antibiotics were stored at −80 °C and aliquots of such solutions were thawed at room temperature and diluted in CAMH broth before each experiment.

### 3.2. Scanning Electron Microscopy (SEM)

For visualization of *P. a*eruginosa CCIN34519 biofilm*,* overnight broth culture of *P. aeruginosa* CCIN34519 in CAMH broth was diluted in PBS to 1.5 × 10^8^ CFU/mL using 0.5 McFarland equivalence turbidity standards (Thermo Scientific Remel™, Lenexa, KS, USA). The cell suspension was used to inoculate sterile CAMH broth at final bacterial count of 1.5 × 10^6^ CFU/mL and 1.5 mL aliquots of the inoculated broth was distributed in 24-well polystyrene plates (Corning Costar®, Corning, NY, USA). The plates were incubated for 12 h at 37 °C without shaking. After incubation, the supernatant was carefully decanted and the wells were washed twice with 2 mL PBS to remove the unattached or loosely attached bacterial cells. Fixation of *P. aeruginosa* CCIN34519 biofilm was conducted at 4 °C with 10% glutaraldehyde (Sigma-Aldrich) for 24 h. Subsequently, the wells were rinsed with a graded series (30%, 50%, 70%, and 100% v/v) of ethanol (Fisher Scientific, Waltham, MA, USA) for dehydration of biofilm specimens and the wells were then entirely air dried prior to SEM examinations [59]. The photo was captured using variable pressure JEOL scanning electron microscope (Model JSM-6490LV, Peabody, MA, USA) outfitted with a tungsten filament of 10 kV accelerating voltages and chamber variable pressure from 60–70 Pa. The adjustable pressure operation enables lower vacuum to exist at the sample chamber. This feature makes it possible to directly observe frozen or non-conductive specimens. The variable pressure chamber of JSM-6490LV also provides a positively ionized gas immediately above the specimen that in turn dissipates the negative charges that accumulate on the sample surface during scanning and interfere with image acquisition. In view of that, non-conductive biofilm specimens can be imaged without application of a conductive gold coating.

### 3.3. Real-Time Monitoring of P. aeruginosa CCIN34519 Biofilm

A previously described *in vitro* system for real-time monitoring of biofilm growth/inhibition [60] was utilized with minor adaptations to assess the antimicrobial effects of as single antibiotics as well as in combinations with zinc sulfate (2.5 mM) against *P.* *aeruginosa* CCIN34519 biofilm. Initial log phase inocula (1 × 10^6^ CFU/mL) of *P.* *aeruginosa* CCIN34519 cultures were seeded (150 µL/well) into 100-well polystyrene honeycomb plates (Growth Curves USA, Piscataway, NJ, USA). *P.* *aeruginosa* CCIN34519 biofilms were allowed to develop in the incubator over 12 h at 37 °C. After incubation, each well of the honeycomb plates were carefully rinsed twice with 150 µL sterile saline for removal of planktonic cells using digital multichannel pipettor adapted for this system. Cation-adjusted Mueller Hinton II (CAMH) broths supplemented with two-fold escalating concentrations of ciprofloxacin, levofloxacin, moxifloxacin, norfloxacin, ertapenem, ceftriaxone, gentamicin, and tobramycin as single antibiotics as well as in combinations with zinc sulfate (2.5 mM) were prepared and transferred to the established *P.* *aeruginosa* CCIN34519 biofilm (250 µL/well) in the honeycomb plates. Plates containing treated, untreated *P.* *aeruginosa* CCIN34519 biofilm, and negative controls were then placed in the preheated incubating chamber of the Bioscreen C which was programmed to maintain a temperature at 37 °C without shaking. Growth control wells of *P.* *aeruginosa* CCIN34519 biofilm treated with CAMH and CAMH spiked with zinc sulfate (2.5 mM) were also implemented in each experiment. Furthermore, negative controls containing CAMH only, CAMH spiked with zinc sulfate (2.5 mM), and CAMH broths supplemented with the above mentioned antibiotics as single agents as well as in combinations with zinc sulfate (2.5 mM) without the biofilm were also involved in the experiments to assure the sterility and stability of their optical density readings overall during 96 h of the incubation period.

In real-time, *P.* *aeruginosa* CCIN34519 biofilm growth/inhibition was monitored using the automated technology of Bioscreen C (Growth Curves USA). The Bioscreen C monitors microbial growth through determining the optical densities in each well. Alterations in the optical densities due to the microbial growth or lysis from the biofilm were measured kinetically with an advanced photometrical technology of the Bioscreen C. A wide-band filter with spectrum range of 420–580 nm was used to measure the optical densities in all experiments. This filter has been utilized in the biofilm research because its sensitivity is not affected by the color transformation resulting from the growth of *P.* *aeruginosa* biofilm [60]. Each biofilm growth curve was repeated three times and the optical density values over 96 h were robotically recorded at 1 h intervals using EZExperiment software (Growth Curves USA) and exported to spread sheets for processing and graphical presentation. Minimum inhibitory concentration (MIC) of the biofilm was defined as the antibiotic concentration at which the optical density remains at the baseline (0.2–0.3) at the 24 h time point, while mutant prevention concentration (MPC) was reported as the antibiotic concentration that prevents growth and development of the resistant subpopulations from the biofilm at 96 h time point [60,61].

## 4. Conclusions

This study demonstrated a heterogeneous range of anti-biofilm efficacies of the antibiotics in combination with zinc sulfate (2.5 mM) against *P. aeruginosa* CCIN34519 biofilm with potential synergisms in cases of fluoroquinolones (ciprofloxacin, levofloxacin, moxifloxacin, and norfloxacin) and carbapenem (ertapenem) as demonstrated by the reduction of their MICs and MPCs. On the contrary, considerable antagonisms were observed with cephalosporin (ceftriaxone) and aminoglycosides (gentamicin, and tobramycin) as shown by the increment of their MICs and MPCs. Further deliberate *in vivo* investigations for the promising synergisms are required to evaluate their therapeutic potentials for treatment of UTIs caused by *P. aeruginosa* biofilms.

## Abbreviations

CLSI: Clinical and Laboratory Standards Institute
MIC: Minimum Inhibitory Concentration
MPC: Mutant Prevention Concentration
NAUTICA: North American Urinary Tract Infection Collaborative Alliance
OTC: Over The Counter
*P. aeruginosa*: *Pseudomonas aeruginosa*
SEM: Scanning Electron Microscope
UTI: Urinary Tract Infection
